# The role and effectiveness of School-based Extra-Curricular Interventions on children’s health and HIV related behaviour: the case study of Soul Buddyz Clubs Programme in South Africa

**DOI:** 10.1186/s12889-021-12281-8

**Published:** 2021-12-11

**Authors:** Lebohang Letsela, Michael Jana, Rebecca Pursell-Gotz, Phinah Kodisang, Renay Weiner

**Affiliations:** 1 Soul City Institute for Social Justice, 1 Newtown Avenue, Killarney, Johannesburg, South Africa; 2 School of Social Sciences, University of the Witwatersrand, 1 Jan Smuts Ave, Johannesburg, South Africa; 3 Research & Training for Health & Development, 9 Lurgan Road Parkview, Johannesburg, South Africa

**Keywords:** Extra-curricular interventions, Children, HIV prevention, South Africa

## Abstract

**Background:**

HIV education targeting children and adolescents is a key component of HIV prevention. This is especially important in the context of increasing HIV prevalence rates among adolescents and young people. The authors sought to examine the role and effectiveness of an extra-curricular school based programme, Soul Buddyz Clubs (SBC) on HIV knowledge, attitudes, behaviours and biomedical outcomes.

**Methods:**

This paper employs a mixed methods approach drawing on data from independent qualitative and quantitative sources. Secondary data analysis was performed using survey data from a nationally representative sample that was restricted to 10-14 year-old males and females living in South Africa. Ten focus group discussions and ten in-depth interviews conducted with SBC members and facilitators from 5 provinces, as part of a process evaluation are used to triangulate the effectiveness of SBC intervention.

**Results:**

The analysis of survey data from 2 198 children indicated that 12% of respondents were exposed to SBC with 4% reporting that they had ever belonged to a club. Children exposed to SBC were more likely to be medically circumcised (AOR 2.38; 95%CI 1.29 -4.40, p=0.006), had correct HIV knowledge (AOR 2.21; 95%CI 1.36 – 3.57, p<0.001) and had less HIV stigmatising attitudes (AOR 0.54; 95%CI 0.31-0.93, p=0.025), adjusting for age, sex, province and exposure to other media – in comparison to those not exposed. Propensity Score Matching findings were consistent with the regression findings. Qualitative findings also supported some of the quantitative results. SBC members reported having learnt about HIV prevention life skills, including condom use, positive attitudes towards people living with HIV, and alcohol abuse.

**Conclusions:**

Participation in SBC is associated with accessing biomedical HIV prevention services, specifically MMC, correct HIV prevention knowledge and less HIV stigmatizing attitudes. This paper demonstrates the effectiveness of a school-based extracurricular intervention using a club approach targeting boys and girls ages 10-14 years on some of the key HIV prevention biomarkers as well as knowledge and attitudes. The article suggests that extra-curricular interventions can form an effective component of school-based comprehensive sexuality education in preventing HIV and promoting medical male circumcision.

**Supplementary Information:**

The online version contains supplementary material available at 10.1186/s12889-021-12281-8.

## Background

In Eastern and Southern Africa, evidence suggests that adolescents (10-19 years) and young people (10-24 years) are still heavily affected by HIV/AIDS. In 2017, adolescents and young people accounted for 37% of all new HIV infections and 15% of all people living with HIV [1]. Overall, AIDS-related deaths have also increased by 50% among adolescents and young people between 2005 and 2017, despite the overall number of AIDS-related deaths declining by 48% during the same period [1].

In South Africa, approximately 260,000 children aged 0 to 14 years lived with HIV in 2018. HIV prevalence among young women (15-24 years) was significantly higher (11.3%) than among young men (3.7%), signifying the vulnerability of girls to the HIV pandemic [2]. This HIV burden among adolescents and young people comes in the context of limited access to HIV and Sexual and Reproductive Health Rights (SRHR) services among this age group [1]. Any effort aimed at addressing HIV, therefore, needs to target this age group if it is to achieve milestones such as the Sustainable Development Goal (SDG) of ending the HIV epidemic by 2030 [3].

For over two decades, the Department of Basic Education in South Africa has been implementing HIV education in schools through various interventions, notably the institutionalisation of Life Skills and Life Orientation as part of the curriculum [4]. The continued increase in HIV incidence among young people, however, indicates complexities in these interventions in addressing HIV risk among children and young people [5]. Visser has indicated that for school-based HIV prevention strategies to be effective, they need to go beyond educating the individual to understanding young people’s sexual risk behaviour and the underlying drivers for these behaviours [5]. This includes addressing interpersonal and community factors such as improving child-parent communication, peer group norms, addressing substance abuse, and shifting unequal gender norms. This is consistent with behaviour change theories within the ecological approach, such as social identity theory, which emphasizes the importance of social norms in determining behaviour change [5–7]. Child-focused interventions that complement curriculum-based interventions while guided by these social and behaviour change theories therefore have the potential to tackle these drivers of adolescent behaviours. Save the Children International’s advice when programming for children is that it is imperative to put the child at the centre [8]. There is a growing recognition that as children transition into adulthood, especially in high-risk situations such as HIV/AIDS, they need safe spaces where they can communicate, socialize, learn and practice life skills and receive psychosocial support [9–13].

The Soul Buddyz Clubs (SBC) programme is one such programme that addresses interpersonal and community factors and gives voice to and promotes action by and for children’s health and well-being. Soul Buddyz Clubs are school-based and target primary school preteens and young adolescents in poor communities based in rural and peri-urban areas, commonly referred to as Townships and Informal settlements in South Africa. By 2020, over 8000 SBC clubs, that reached approximately 190 000 pupils, had been established across schools in South Africa over the past eighteen years. The clubs emerged from a health and development communication perspective with the strategic goal of empowering children through education and life skills, increasing their efficacy to deal with adolescent sexuality and HIV prevention, and becoming agents of change, embedding the principle of child participation. As an extracurricular activity, SBC activities are supported by volunteer facilitators (educators at schools where the clubs are implemented) and are aimed at increasing knowledge and skills about health and wellbeing and strengthening the existing Life Orientation and Life Skills curriculum taught in schools [4, 14, 15]. According to Centers for Disease Control and Prevention (CDC), comprehensive school health is based on a curriculum that includes an array of topics such as personal, family, community, consumer and environmental health; sexual health education; mental and emotional health; injury prevention and safety; nutrition; prevention and control of disease; and alcohol, tobacco and other drugs – that are delivered by well-trained teachers [16, 17].

The role of teachers as facilitators of the Soul Buddyz Clubs has been fundamental to their success and sustainability since 2003, when clubs were initiated. The objectives of the clubs are to provide learners with edutainment that allows them to have fun while learning, promoting positive attitudes and behaviours that relate to HIV, such as self-efficacy for safer sexual behaviour, condom use, gender-based violence and alcohol and drug use [18]. Club activities range from regular after-school club meetings, discussions and debates on important themes, competitions and projects on topics of interest including HIV/AIDS testing, treatment and stigma and discrimination; sexual and reproductive health; healthy lifestyle (nutrition, diet and physical activity); medical male circumcision (MMC); Tuberculosis, bullying; and community participation. Facilitators are provided with training on the programme that is premised on the comprehensive school health education guidelines, as well as additional resources and materials, including booklets, magazines, and posters, which are used to steer these discussions [14]. The clubs promote action by children, facilitating their ability to organise themselves and be agents of change for children’s issues.

Existing literature on school-based programmes aimed at improving the sexual and reproductive health of adolescents indicates that school- or community-based sexuality and HIV/AIDS education programmes increase knowledge, may reduce the number of sexual partners and increase condom and contraceptive use [19–21]. There are also reported improvements in attitudes towards people living with HIV/AIDS, intention to abstain or use a condom and self-efficacy for condom use; increase in talking to others (friends, peers/parents/boyfriend/girlfriend) about sexual risk and delay sexual debut [20, 22]. Contrary to fears expressed by parents, school-based sexuality education programs do not lead to early sexual debut or increased sexual activity [23–26]. A review by Mason-Jones et al. revealed that combined (sexual and reproductive) educational and incentive-based programmes had a positive effect on sexually transmitted infections (STIs) (herpes simplex virus infection) [27]. Ross et al. indicated that incentive-based interventions are likely to reduce adolescent pregnancy [22].

While there are several programmes for adolescents in South Africa (e.g., DREAMS; YOLO, loveLife), there is a gap in programmes targeting 10–14-year-olds. SBC is one of the few established programmes that targets this age group, a key age to target for prevention amidst the argument that even though the majority have not yet begun sexual activity, there is growing evidence that pubescents are exposed to sexual risk and vulnerability at a younger age. For example, the HSRC survey found that sexual debut < 15 years increased from 8.5% in 2008 to 13.6% in 2017. This is the case for both males and females: from 11.3% to 19.5% for males and 5.9% to 7.6% in females [28]. Furthermore, sexual coercion associated with sexual debut has been an issue of concern, as highlighted in a study in KwaZulu-Natal by Maharaj & Munthree, which found that nearly 46% of all adolescent girls and young women (14-24 years) reported that their first sexual encounter had been coerced and that this group was more likely to have had an STI or unintended pregnancy [29].

There is paucity of evidence on the effectiveness of extracurricular school-based programmes in promoting HIV prevention. A soccer-based programme implemented in Uganda and Zimbabwe that aimed to promote voluntary medical male circumcision (VMMC) among schoolboys reported increases in male circumcision (10% and 26%) [30, 31], as well as almost three times the increased likelihood of performing VMMC [32]. Studies that documented support required by adolescents affected by HIV/AIDS in schools revealed that within the context of high rates of psychological, behavioural and emotional problems among youth, support by teachers’ peers and the general school environment amidst experiences of discrimination, social exclusion, labelling and bullying in school was not adequate [33, 34].

The objective of this paper is to investigate the role and effectiveness of an extracurricular school-based programme on HIV knowledge, attitudes and practices, as well as biomedical outcomes (HIV testing and MMC) among children. The paper seeks to demonstrate the value of participatory interventions that are child-centred and provide an open and interactive environment while addressing HIV risk and life skills.

## Methods

The paper uses a mixed methods approach drawing on and integrating data from independent quantitative and qualitative sources.

### Quantitative research design and sample

The quantitative data source was the fifth national survey - the South African National HIV Prevalence, Incidence, Behaviour and Communication Survey (SABSSM V, 2017), on which secondary analysis was performed to measure behavioural outcomes of the SBC programme over time. This survey was a nationally representative, cross-sectional survey of all ages living in South Africa conducted between 2017 and 2018 by the Human Science Research Council (HSRC). One thousand small area layers (SALs), as defined by Statistics South Africa, were randomly selected from a total of 84 907 SALs (STATS-SA, 2017). Furthermore, to enrol individuals into the survey, 15 households from each SAL were selected using systematic sampling. A total of 11,776 households were ultimately included in the sample of which 82.2% of household heads consented to participate in the survey. All household members were eligible for participation in the survey. Dried Blood Spot (DBS) specimens were collected from all consenting survey respondents and samples were tested for HIV antibodies using an algorithm [28, 29]. For this paper, the authors used data for the 10- to 14-year-old age group to perform analyses.

The survey questionnaire, information sheets and informed consent forms were translated from English into the 10 other South African official languages: Afrikaans, isiZulu, isiXhosa, Sesotho, Setswana, Sepedi, SiSwati, Xitsonga, Tshivenda, and IsiNdebele) and piloted prior to the main fieldwork taking place [28].

Soul City Institute for Social Justice was a partner on the HSRC survey and measurement of its programmes included the following exposure variables: overall exposure to the Soul Buddyz Clubs Programme was measured through a combination of the following questions from the survey: “*Have you ever belonged to a Soul Buddyz Club?” and if yes, for how long?* All respondents, regardless of their Soul Buddyz Club membership, were asked, “*Have you read any of the following Soul Buddyz Club booklets in the past 12 months*?” (See list of booklets shown in Table 1).

**Table 1:** Soul Buddyz Club Materials

SBC Material	Topic of focus
The Zone	Sexual and Reproductive Health: HPV vaccine
	Healthy living: healthy eating
	Medical Male Circumcision
	Safety on the road
	Relationships, learning about the brain, Tobacco
	GBV, bullying, alcohol, personal development (saving/ starting business)
Unit Guides	Alcohol: alcohol & drug free school
	HIV stigma reduction, acceptance, and treatment adherence
Posters	TB, alcohol (alcohol free schools), HIV (stigma), drug usage

### Survey data analysis

The analysis was restricted to and based on a weighted sample size of 2 198 10- to 14-year-olds. The results are reported by sex, age, self-reported race (Black African, Coloured, Indian, White and other), geographical type (urban, traditional rural areas, or commercial farming communities) and by each of the nine provinces in South Africa. Population weights based on province, sex, age, and race were used to account for the complex survey design and non-response, and weighted data analysis was implemented using “svyset” in Stata MP/14.0. Weights were applied according to the SABSSM V schedule to account for non-respondents and to mitigate the selection bias inherent in the sampling procedure [28]. Bivariate and multivariate analyses were conducted to determine the impact of exposure to SBC on HIV knowledge, behaviours and biomedical indicators (HIV testing and MMC). Specifically, the following outcomes were measured: sexual behaviours (ever having sex, condom use at first sex), HIV knowledge and stigmatizing attitudes (described on Table 2), HIV antibody testing, HIV status and medical male circumcision uptake. Bivariate (chi-square test of significance for frequency tables and cross tabulations) and multivariate analyses included logistic regression and propensity score adjusted models (PSMs). For PSM, sociodemographic variables such as age, sex, level of education (grade), province and exposure to other media were used as control and matching variables. The results of the final logistic regression models are reported using adjusted odds ratios (AORs) and 95% confidence intervals (CIs). The exposure variable was fitted in all the models.

**Table 2:** Outcome measures

Outcome	Questions	Response options	Notes
**Knowledge of HIV**	Measured using a knowledge scale which combined a set of questions: Knowledge of HIV and AIDS: “Can AIDS be cured?” “Can a person reduce the risk of HIV by having fewer sexual partners?” “Can a healthy-looking person have HIV?” “Can HIV be transmitted from a mother to her unborn baby?” “Can the risk of HIV transmission be reduced by having sex with only one uninfected partner who has no other partners?” “Can a person get HIV by sharing food with someone who is infected?” “Can a person reduce the risk of getting HIV by using a condom every time he/she has sex?” “Can medical male circumcision reduce the risk of HIV infection in males?” “Are there medicines that people with HIV or AIDS can take to help them live longer?”	Yes/ No/ Don’t know	A continuous HIV knowledge variable was created from 10 questions that looked at HIV knowledge, excluding knowledge of PMTCT. Values for the knowledge variable ranged from -9 to 19. A categorical variable for HIV knowledge was created with the following scores: 0 = no / poor knowledge with misconceptions (-9 to 0) 1 =correct knowledge (1 to 19)
**HIV stigmatizing attitudes**	A continuous HIV stigma variable was created from six questions that looked at attitudes towards people living with HIV: Questions relating to people living with HIV/AIDS: “If you knew that a shopkeeper or food seller had HIV, would you buy food from them?” “Would you be willing to care for a family member with AIDS?” “If a teacher has HIV but is not sick, he or she should be allowed to continue teaching?” “Would you be willing to share food with someone who has HIV or AIDS?” “Are you comfortable talking to at least one member of your family about HIV/AIDS?” “Would you play with someone who has HIV or AIDS?”	Yes/ No/ Don’t know	Values for the stigma variable ranged from 0 to 6. A categorical variable for HIV knowledge was created with the following scores: Has stigma=0 to 2 (low score) No stigma= 3 to 6 (high score)
**Medical male circumcision**	Some men are circumcised. Have you been circumcised? “Who performed the circumcision?”.	Yes/No Medical Doctor/ or Nurse/ Spiritual or religious leader/ Traditional circumciser/ Other/ Don’t know	Restricted to men who have been circumcised A categorical variable for medical male circumcision was created for: 1= performed by doctor/nurse 0= not performed by doctor/nurse
HIV testing	“Have you ever had an HIV test?” “How long ago did you have your most recent HIV test?”	Yes /No/ Don’t Know	A categorical value was created for “how long ago did you go for your most recent test”: 12 months or less (Recent) and more than 12 months (12 months)
**HIV prevalence**	Measured HIV seroprevalence	Negative/Positive	

### Qualitative research methods

Qualitative data were generated from a process evaluation study that was conducted by a team of researchers led by one of the co-authors (Dr Michael Jana) through an independent research organisation called Research and Training for Health Development (RTHD). Qualitative data was collected with SBC members, programme implementers and stakeholders in five provinces in South Africa in 2018. The provinces include Eastern Cape, Gauteng, KwaZulu Natal, Mpumalanga and Western Cape.

### Qualitative data collection and sample

Interviews were conducted with ten SBC facilitators (2 per province) using a structured interview guide. A total of 10 focus group discussions (FGD) with SBC boys and girls aged 10 to 14 years across the five provinces were held to understand the quality of implementation, lessons learned and whether the objectives were met. FGDs with children were conducted in the local language of that specific province (vernacular included isiXhosa, isiZulu, Sesotho and Afrikaans). Each FGD consisted of about 10 to 12 people. Both the FGD and interview guides were developed by Dr Michael Jana and his team.

Boys and girls were separated in some groups and mixed in others to gauge whether that would have an effect on their responses. Findings, however, do no show marked differences in responses. The use of FGDs including participatory methods, was prioritised for SBC members to explore their experiences of the club and social norms related to social and behaviour change.

As part of the FGD participatory activities, club members were asked to draw as a group what they did during club meetings, and what they had learnt. They were also asked to describe their drawing during the focus group discussion, as well as elaborate on the types of activities they had undertaken in their school and community.

### Data analysis

Qualitative data were analysed using thematic analysis in ATLAS.ti and triangulated by comparing emerging themes as well as related findings across the data sources (FGD and interview transcripts). Two people were involved in the qualitative analysis and codes were generated inductively by reading through the transcripts and coding the essence of the responses before grouping similar codes into sub-themes and themes as suggested by Patton [36] and Neuman [37]. The overlapping or common themes were noted as cross-cutting findings while maintaining the other themes as either audience-specific findings “according to particular FGD or interview province/age” or findings that were not prominent. The emerging themes were then written up in narratives in line with the research questions and supported by illustrative direct quotations and children’s drawings. This paper uses the qualitative findings on the effectiveness of the SBC program in an effort to triangulate the quantitative findings.

### Ethical clearance

Ethical approval was received from the HSRC Ethics Committee (Protocol no 4/18/07/18) to undertake qualitative research with learners in schools. The survey was approved by the HSRC Research Ethics Committee (REC: 4/18/11/15) and reviewed in accordance with CDC human research protection procedures.

## Results

### Reach and Relevance

A total of 39,132 participants of all ages of which 3,818 (9.8%) were adolescents from 11,776 households were eligible to participate in the main study. The weighted sample using the “svyset” in Stata MP/14.0 yielded a total of 2 198 participants, ages 10-14 years, that informed the quantitative secondary data analysis that the results of this paper are based on - the sociodemographic characteristics of the sample are presented in Table 3.

**Table 3:** Socio-demographic characteristics of the SBC members vs non members

	Exposed to SBC		Unexposed to SBC		Total		P-value
	No.	%	No.	%	No.	%	
**Age**							
10 - 12 years	78	30%	746	41%	832	40%	0.004
13-14 years	182	70%	1073	59%	1247	60%	
**Total**	**260**	**100.0**	**1819**	**100.0**	**2079**	**100.0**	
**Sex**							
Male	104	40%	928	51%	1034	49.7	0.01
Female	156	60%	891	49%	1045	50.3	
**Total**	**260**	**100.0**	**1819**	**100.0**	**2079**	**100.0**	
**Population group**							
Black African	229	1	1510	83%	1746	84%	0.27
White	8	0	109	6%	104	5%	
Coloured	18	0	164	9%	187	9%	
Indian/Asian	5	0	36	2%	42	2%	
**Total**	**260**	**100.0**	**1819**	**100.0**	**2079**	**100.0**	
**Province**							
Eastern Cape	23	9%	255	14%	291	14%	0.07
Free State	18	7%	109	6%	125	6%	
Gauteng	81	31%	364	20%	437	21%	
KwaZulu-Natal	52	20%	400	22%	457	22%	
Limpopo	29	11%	236	13%	249	12%	
Mpumalanga	10	4%	109	6%	125	6%	
North West	23	9%	127	7%	146	7%	
Northern Cape	5	2%	36	2%	42	2%	
Western Cape	18	7%	182	10%	208	10%	
**Total**	**260**	**100.0**	**1819**	**100.0**	**2079**	**100.0**	
**Grade**							
Grade 1	5	2%	18	1%	20	1%	0.007
Grade 2	5	2%	54	3%	41	2%	
Grade 3	5	2%	143	8%	143	7%	
Grade 4	31	12%	286	16%	327	16%	
Grade 5	59	23%	447	25%	511	25%	
Grade 6	52	20%	411	23%	470	23%	
Grade 7	80	31%	321	18%	409	20%	
Grade 8	21	8%	107	6%	123	6%	
**Total**	**258**	**100.0**	**1786**	**100.0**	**2044**	**100.0**	

The mean age of the respondents was 13 years (standard deviation: SD = 0.82). There were slightly more males (51%) than females (49%). There was a significant difference in age with more 13- to 14-year-olds (70%) than 10–12-year-olds (30%) (p=0.004) as well as sex (p=0.01) between those exposed and not exposed to SBC (60% females and 40% males). A quarter were in Grade 5. The majority of respondents exposed to SBC were attending Grade 5 to 7 (23% in Grade 5 and 20% in Grade 6), with significantly more Grade 7 (last year of primary school) participating in SBC compared to those not exposed (18%) (p = 0.007).

The survey indicated that 12% of the 10- to 14-year-old respondents were exposed to the Soul Buddyz Clubs programme, which represents 354 256 children, 314 179 of which were exposed to the SBC booklets. More respondents aged 13-14 years (8.9% N= 248 259) than 10-12 years (3.8% N = 105 998) were reached with the programme. Reach was slightly higher among females (7.6% n= 211 997) than males (5.1% n = 142 261). Four percent of respondents who were exposed to the programme, equating to 108 256 people, reported that they had ever belonged to a SBC. Most respondents who had ever belonged to a SBC indicated that they were part of a SBC for one year (54% N = 55 490).

**Fig. 1:**
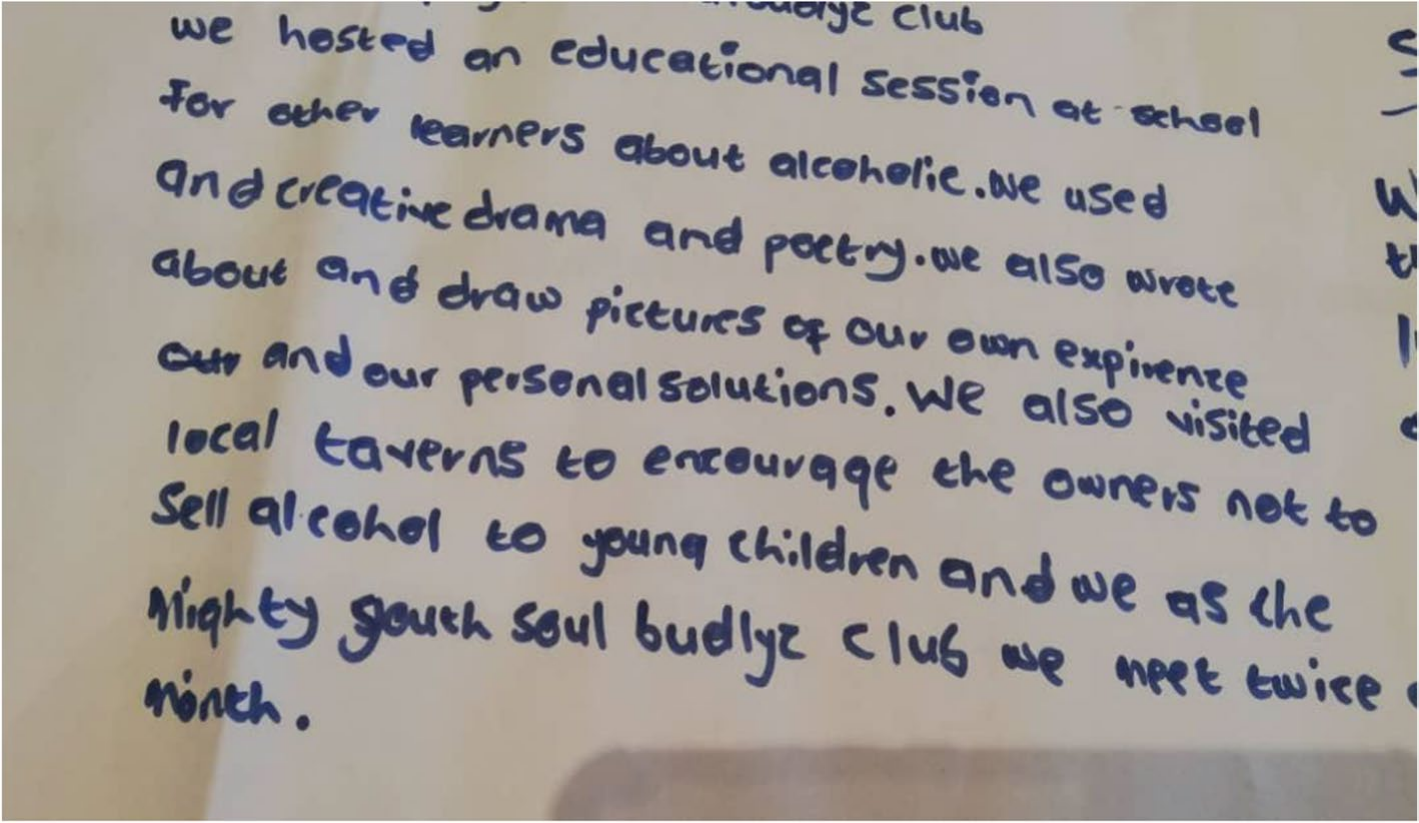
SBC FGD with girls ages 10-12 years, Gauteng, February 2019

There was strong qualitative evidence supporting the relevance of the programme. Children live in a context where there is drug and alcohol abuse, sexual abuse, lack of communication between children and their parents, HIV and AIDS and exposure to unsafe places. By speaking directly to these issues, SBC was deemed to be relevant, as illustrated in Fig. 1 – a quote from an FGD with children:

Figure 1 above illustrates the perceptions of how SBC activities responded to the health environment of the children who participated in the FGDs. The picture talks about SBC school sessions to discuss the effects of alcohol and visits to local taverns to discourage the sale of alcohol to children. The quotation below further illustrates the relevance of SBC:

*We talk about having to be responsible towards others. We talk about everything other people have done to us. We go to the garden every day and talk about soul buddyz. Every Tuesday they give us homework to do. We have learned a lot of things from Soul Buddyz. - SBC FGD with mixed gender, ages 10- to 12-year-olds, Gauteng, January 2019*

Key aspects of success and sustained participation include that the clubs are child-led, provide peer support, are contextualised to the issues at hand and engage a passionate facilitator that is approachable and recognises the contribution of young people to their own development and as change agents in their own lives. Facilitators used materials for club activities that are varied in nature as highlighted by the quotation below:

*We have a Soul Buddyz Club guide that determines the activities; but there are others that come from the creativity of the learners, for example dancing, singing... There is an activity where they have to be aware of their environment, so they identified students who are in need and alerted us (facilitators) and we took the matter to the principal and had an appointment with the social workers and the social worker took it up.... we also helped them with food – there is a school feeding programme here; another issue was that there was a granny guardian without (social) grant... - SBC Facilitator, Mpumalanga Province, November 2018*

The relationships between facilitators and club members were also identified as a critical success factor. Where club members felt that they could trust facilitators, they felt that they could disclose things to the facilitator, and they would get help. Club members had the following to say about facilitators:

*I like them – they understand us.- SBC FGD with mixed gender, ages 10- to 12-year-olds, Western Cape, February 2019*

## Knowledge and psychosocial skills outcomes

### HIV knowledge

Seventy-three percent of the respondents demonstrated correct HIV prevention knowledge measured using a knowledge scale that combined a set of questions on knowledge of HIV/AIDS. A continuous HIV knowledge variable was created from 10 questions that looked at HIV knowledge. There was very strong evidence of an association between HIV knowledge and exposure to SBC, with more respondents who were exposed to SBC reporting having correct knowledge (87%) in comparison with those not exposed to the programme (75%) (p<0.0001) (see Table 4). This finding was supported by the qualitative data with SBC members reporting that Soul Buddyz had improved their knowledge of HIV, including how to protect themselves from contracting HIV, the importance of consistent condom use and identifying myths and misinformation about HIV such as that you can see by looking at someone if they have HIV as illustrated in the quotes below.

*I’ve learnt about sex and not to go too deep – not to have a sexual relationship. - SBC FGD with mixed gender, ages 10- to 12-year-olds, Western Cape, February 2019*

*As a girl, I learned not to be sexually active and to protect myself from boys (by not having sex or using condoms if I have sex). - SBC FGD with mixed gender, ages 10- to 12-year-olds, Western Cape, February 2019*

*The book (Soul Buddyz Booklet) tells us about looking after ourselves, gender and protecting ourselves from diseases like HIV and AIDS. - SBC FGD with boys, ages 10-12-year-olds, Mpumalanga, November 2018*

**Table 4:** Associations between socio - demographic characteristics, HIV knowledge and stigma, MMC and HIV testing among SBC membership

Correct HIV prevention knowledge	Exposed to SBC		Unexposed to SBC		Total		P-value
	No.	%	No.	%	No.	%	
No	35	13%	452	25%	499	24%	0.0001
Yes	235	87%	1357	75%	1580	76%	
**Total**	**270**	**100**	**1809**	**100**	**2079**	**100**	
**HIV stigmatizing attitudes**							
No stigma	238	89%	1413	79%	1665	81%	0.005
has stigma	29	11%	376	21%	391	19%	
**Total**	**267**	**100**	**1789**	**100**	**2056**	**100**	
**Ever had sex**							
No	260	98%	1754	99%	1996	98%	0.33
Yes	5	2%	18	1%	41	2%	
**Total**	**265**	**100**	**1772**	**100**	**2037**	**100**	
**Condom use at first sex**							
Did not	2	49%	8	46%	11	47%	no p-value
Used condom	3	51%	10	54%	12	53%	
**Total**	**5**	**100**	**18**	**100**	**23**	**100**	
**Medical Male Circumcision**							
No	46	45%	625	68%	674	66%	0.0005
Yes	56	55%	294	32%	347	34%	
**Total**	**102**	**100**	**919**	**100**	**1021**	**100**	
**Recent HIV test**							
No	187	73%	1391	81%	1579	80%	0.02
Yes	69	27%	326	19%	395	20%	
**Total**	**257**	**100**	**1717**	**100**	**1974**	**100**	
**HIV Status**							
Negative	186	98%	1234	97%	1418	97%	0.97
Positive	4	2%	38	3%	44	3%	
**Total**	**190**	**100**	**1272**	**100**	**1462**	**100**	

Soul Buddyz also helped members identify and address myths and incorrect information about HIV and how HIV is acquired. Club members noted that one of the myths they had heard which was corrected through Soul Buddyz was:

*People get AIDS if they walk where someone spit and they have the sickness. - SBC FGD with mixed gender, ages 10- to 12-year-olds, Western Cape, February 2019*

## HIV stigmatizing attitudes

**Fig. 2:**
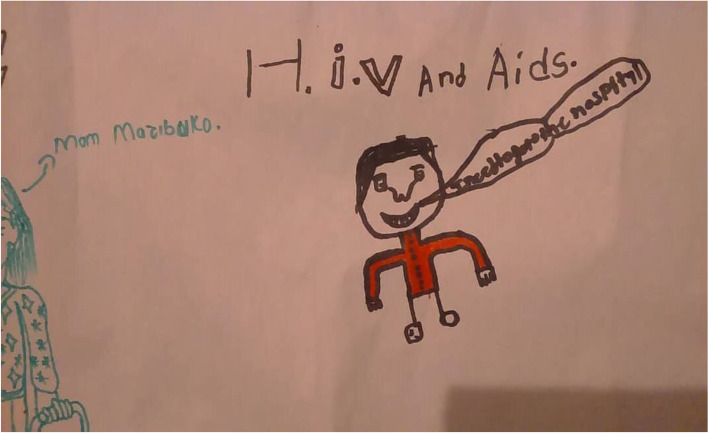
SBC FGD with boys, ages 10- to 12 years, Mpumalanga, November 2018

A total of 20% of teenagers were reported to have stigmatizing attitudes measured using a stigma scale which combined a set of questions on HIV/AIDS stigma. A continuous HIV stigma variable was created from six questions that looked at attitudes towards people living with HIV (see Table 2). There was strong evidence of an association in reporting stigmatizing attitudes between those exposed to SBC and those unexposed (p =0.005) with more respondents exposed to SBC reporting not having stigmatizing attitudes (89%) compared to those not exposed (79%) (See Table 4). Club members from the qualitative study reported having learnt how people can be affected by stigma and misinformation. See Figure 2 for a drawing depicting a discussion on HIV/AIDS and the importance of going to the hospital for regular check-ups and treatment.

*Yes, some children are born HIV positive, and when other children find out about their status, they do not want to play with them. We play with those children because they are like the rest of us. We also play with them because we will also need help one day. - SBC FGD with mixed gender, ages 10- to 12-year-olds, KZN, January 2019.*

## Risky sexual behaviour

### Sexual activity and condom use

Some 1.5% respondents reported having ever had sex. There was an insignificant association between sexual activity, measured by “ever having sex” and exposure to SBC p =0.33 (see Table 4). Some 53% respondents who reported ever having sex, reported having used a condom the first time they had sex. There were too few observations to measure the association between exposure to SBC and condom use at first sex. Qualitative findings further highlighted that children learned about safe sex and the use of condoms from SBCs.

*If someone has HIV, you won’t know just by looking at them. If you can’t get tested then rather just use condoms. Yes, safe sex. They teach us about that in Soul Buddyz. - SBC FGD with females, ages 13- to 14-year-olds, Western Cape, January 2019*

## Access to biomedical intervention

### Medical Male Circumcision

Thirty-four percent (34%) of males reported having had MMC performed by a doctor. There was very strong evidence of an association between medical male circumcision and exposure to SBC (p==0.0005). More males exposed to SBC reported being medically circumcised than those not exposed (55% versus 32%) (see Table 4).

### HIV testing and status

A total of 20% of the respondents reported having been tested for HIV. There was evidence of an association between HIV testing and exposure to SBC (p=0.02). More adolescents exposed to SBC (27%) reported taking an HIV test than those not exposed to SBC (19%). Moreover, some 3% of respondents tested positive for HIV – there was no statistical significance in testing HIV negative between those exposed to SBC compared to those unexposed to SBC (p=0.97) (See Table 4).

## Multivariate analysis findings: The relationship between risky sexual behaviours, HIV status, MMC and exposure to SBC

The multivariate regression analysis models presented in Table 5 reveals that after controlling for age, sex, level of education, province and exposure to other media, SBC exposure was significantly associated with medical circumcision (AOR 2.38; 95% CI 1.29 -4.40, p=0.006), HIV knowledge (AOR 2.21; 95% CI 1.36 – 3.57, p<0.001) and less stigmatizing attitudes (AOR 0.54; 95% CI 0.31-0.93, p=0.006). Addressing selection bias in trying to estimate the effect of the intervention, we conducted a PSM (see Table 6) – the PSM findings are consistent with the regression findings showing that the probability of having stigmatizing attitudes is -0.09 percentage point (0.9% point) reduced, and a 0.26% percentage point increase in doing MMC and 0.102% percentage point increase in having correct HIV prevention knowledge, in the exposed as opposed to those unexposed*.*

**Table 5:** Adjusted odds ratio for behavioural and biomedical outcomes in exposure to SBC estimated by logistic regression

	Unadjusted Odds ratio	95% CI	Adjusted Odds ratio	95% CI	P-value	Sample size
Correct HIV prevention knowledge	2.35*	(1.49 - 3.68)	2.21	1.36 - 3.57	0.001	2 079
HIV stigmatizing attitudes	0.47***	(0.27 - 0.81)	0.54	0.31 - 0.93	0.025	2 056
Medical Male Circumcision	2.59*	(1.49 - 4.52)	2.38	1.29 - 4.40	0.006	1 004
Recent HIV test	1.57**	(1.07 - 2.32)	1.48	0.97 - 2.25	0.06	1 974
Ever had sex	1.82**	(0.53 - 6.31)	1.44	0.42 - 4.88	0.56	1 916
Ever consuming alcohol	1.67**	(0.92 - 3.05)	1.47	0.79 - 2.71	0.22	2 078
HIV Status	0.98**	(0.31 - 3.10)	0.95	0.29 - 3.11	0.93	1 462

Controlling for all confounders except for MMC that excluded gender (age, province, exposure to media)

*p=0.001

**p>0.05

***p<0.01

**Table 6:** Propensity score matching treatment effect of exposure to SBC for behavioural and biomedical outcomes

Average treatment effect		% frequency	Coefficient	95% CI	P value
Correct HIV prevention knowledge	SBC exposure	15%	.102	0.101 - 0.103	<0.0001
	unexposed	85%			
Medical Male Circumcision	SBC exposure	16%	.229	0.226 - 0.231	<0.0001
	unexposed	84%			
HIV stigmatizing attitudes	SBC exposure	7%	-.092	(-)0.093 - (-)0.091	<0.0001
	unexposed	93%			
Recent HIV test	SBC exposure	17%	.056	0.054 - 0.057	<0.0001
	unexposed	83%			
HIV Status (positive)	SBC exposure	13%	-.0040296	(-)0.004 - (-)0.003	<0.0001
	unexposed	87%			
Ever had sex	SBC exposure	21%	.008	0.007 - 0.008	<0.0001
	unexposed	79%			
Ever consuming alcohol	SBC exposure	19%	.011	0.010 - 0.012	<0.0001
	unexposed	81%			

Adjusting for age, sex, province and exposure to other media, adolescents exposed to SBC have a positive but insignificant association with HIV testing (AOR 1.48 95% CI 0.97-2.25 p= 0.06) and a negative HIV test outcome with 5% decreased (less) odds of being HIV positive compared to those not exposed (AOR 0.95 95% CI 0.29-3.11, p=0.93).

While teenagers exposed to SBC have 1.44 odds of having had sex compared to those not exposed, this was statistically insignificant (AOR 1.44 95% CI 0.42-4.88; p=0.56). There were too few observations to draw any significant results for condom use at first sex among those exposed to SBC compared to those unexposed. In summary, this analysis has demonstrated that being a Soul Buddyz Club member has a positive effect on biomedical HIV prevention uptake, specifically MMC and HIV testing, HIV prevention knowledge and HIV-related stigma outcomes. Qualitative findings support some of these findings with SBC members indicating having learnt life skills related to HIV prevention through participation in the clubs.

## Discussion

### Key results

This paper has demonstrated the effectiveness of a school-based extracurricular intervention using a club approach targeting boys and girls aged 10-14 years on some of the key HIV prevention biomarkers, as well as knowledge and attitudes as demonstrated by previous studies [20, 22]. This paper contributes to existing evidence on the uptake of MMC in response to an extramural school programme in Southern Africa [31–33]

### Interpretation

The findings are consistent with existing literature that documented the impact of the CSE programme on HIV prevention knowledge. The UNFPA evidence brief on the effectiveness of CSE in preventing HIV reports strong evidence that in-school CSE leads to improved knowledge, increased condom use, decrease in multiple partners, increase in self-efficacy for HIV protection, favourable attitudes to safer sex and delays in initiation of first sexual intercourse [38, 39]. Similarly, there is evidence that school-based sexual and reproductive health programmes improve knowledge and reduce self-reported risk taking behaviour [18–21]. A retrospective study of the Soul Buddyz Club revealed that ex-SBC members are three times more likely to be HIV negative, twice more likely to have one sexual partner and use a condom at first sex, 10 years after participating in the programme [18].

Additionally, a significant body of evidence also shows that CSE not only enables children and young people to develop appropriate attitudes and skills that contribute to safe, healthy and positive relationships but also helps them to reflect on social norms, cultural values and beliefs to better understand and manage their relationships with peers, parents, teachers and other adults in their surroundings [40]. The Soul Buddyz model complements and enhances the school’s Life Orientation and Life Skills curriculum through drawing on volunteer teachers who are passionate about the goals and aims of the programme and undergo specific training in this regard, and implements activities that are based on topics that are underpinned and aligned to international guidelines on sexual health. Furthermore, there is evidence that shows the limited impact of curriculum-based Life Orientation interventions in South Africa on HIV/AIDS among young people for the past 20 years and emphasizes the need for interventions that go beyond educating an individual to address interpersonal and community factors [4]. The SBC Programme is modelled on interpersonal and community factors such as parent-child communication, peer group norms, and addresses substance abuse and gender norms. This paper, therefore, using the case of the SBC programme, demonstrates the potential for extracurricular interventions to complement curricular-based interventions in influencing positive HIV behaviours and outcomes.

The value of SBC is in imparting health and social skills that encourage children to support each other, be agents of social change primarily in their school and increase their sense of self-worth and confidence [13]. This paper has demonstrated an impact on HIV-related stigma, showing that children exposed to the SBC programme have positive and caring attitudes towards people living with HIV. As such this paper contributes knowledge to previous studies that document the inadequate support received by adolescents affected by HIV/AIDS in schools [34, 35].

The programme focuses on topics that are translated into club discussion and activities that respond to the needs of the young people enrolled in the clubs, providing opportunities to address real-life challenges and concerns for these young people. This opportunity has helped club members develop skills and knowledge that have contributed to reducing HIV-related stigma and increased numbers of club members having undergone MMC compared to young people who were not club members. We believe this to be important for two reasons – it means a decline in the number of young boys who may have gone through a traditional circumcision school where there are a number of health risks, and an increase in the number of people who may choose MMC as a means of reducing the risk of HIV infection.

The presented evidence indicates that programmes such as the Soul Buddyz programme, which are rooted in child participation principles and implemented as children’s safe spaces to tackle social issues, are effective in providing an opportunity for pupils to engage in difficult conversations, elicit peer support, develop skills to navigate social situations and enable children to make choices about their own future. Key aspects of success and sustained participation include that the clubs are child-led, provide peer support, are contextualised to the issues at hand and engage a passionate facilitator that is approachable and recognises the contribution of young people to their own development, and as change agents in their own lives [13]. Since under-resourced schools and communities typically lack access to extra-curricular activities, both due to the absence of facilities and resources, Soul Buddyz offers an enriching, fun and educational opportunity for children to belong and participate in positive activities and supplements the existing content of the CSE curriculum taught in school classrooms in a non-threatening way. The clubs have demonstrated themselves to be powerful vehicles for engaging young people aged 10-14 years in solving their own problems, in sharing knowledge about HIV and sexual behaviour, and in discussing social issues which place young people at risk of HIV infection.

## Limitations

The following limitations apply to this paper. The survey conducted by the HSRC was a cross-sectional study design that often cannot determine a temporal relationship. Although respondents were asked if they had belonged to a club in the last 12 months, some interventions such as MMC may have taken place before membership. There were few observations to perform analyses on some of the key programmatic outcomes, notably bullying and teen pregnancy, meant that these outcomes could not be fully assessed.

Moreover, there were positive but insignificant findings on HIV testing and being HIV negative among those exposed to the programme. There was also no impact on condom use at first sex. This lack of impact or significant findings on HIV testing and condom use may be due to the small sample size and/ or the design of the SBC programme and topics covered. For example, there may have not been much emphasis on HIV testing and condom use in the clubs given the age (<14 years) of the SBC members. Continued work specifically focused and appropriate to this age group is required to encourage HIV testing and condom use particularly for sexually active young adolescents.

## Conclusion

There is scant evidence on the effectiveness of extracurricular school-based programmes in promoting HIV prevention, particularly among young people aged 10-14 years. This study fills this gap in knowledge on the role and success of school-based programmes, particularly extracurricular programmes on HIV knowledge, attitudes and practices, as well as MMC among South African children aged 10-14 years who were exposed to the Soul Buddyz Clubs programme. It further builds on the initial work by Schmid et al. [13] showing the unique value of SBC and how the programme has been implemented, which likely impacted observed programme outcomes. Overall, the paper builds on an emerging body of knowledge about the use of school-based extracurricular programmes to work with at-risk youth as part of HIV prevention and education through the use of Social and Behaviour Change Communication. SBC program designers need to consider reviewing and strengthening their messaging on HIV testing, teenage pregnancy, delaying sexual debut, and alcohol consumption. There is a need to design a rigorous evaluation study to appropriately measure SBC-specific outcomes and long-term impact, including bullying and teenage pregnancy. Continued support and implementation of programmes of this nature is essential, given that there are few interventions targeting this age group, which are necessary as they are in the early, delicate, pubescence stage and can mould them into responsible adolescents. More research on how and why these programmes work would also be useful to inform intervention design and implementation.

## Supplementary Information

**ESM 1.**

**ESM 2.**

**ESM 3.**

## Data Availability

The datasets used and/or analysed during the current study are available from the corresponding author on reasonable request.

## Abbreviations

SRHR: Sexual and Reproductive Health Rights
SDG: Sustainable Development Goals
SBC: Soul Buddyz Clubs
CSE: Comprehensive Sexuality Education
GBV: Gender Based Violence
MMC: Medical Male Circumcision
VMMC: Voluntary Medical Male Circumcision
DREAMS: Determined, Resilient, Empowered, AIDS-free, Mentored and Safe
YOLO: You only Live Once
HSRC: Human Sciences research Council
SABSSM V: The fifth South African National HIV Prevalence, Incidence, Behaviour and Communication Survey
FGD: Focus Group Discussion
CDC: Centers for Disease Control and Prevention
UNFPA: United Nations Population Fund
SBCC: Social and Behaviour Change Communication
