# Supplementary material for: The role and effectiveness of School-based Extra-Curricular Interventions on children’s health and HIV related behaviour: the case study of Soul Buddyz Clubs Programme in South Africa
Source: BMC Public Health. 2021 Dec 11;21:2259. doi: 10.1186/s12889-021-12281-8 (PMC8666065; doi:10.1186/s12889-021-12281-8)
Supplement: Supplementary file 1 — ESM 1. [file 12889_2021_12281_MOESM1_ESM.pdf]

Guide for Focus Group Discussions with Children (SBC members) 10 – 12 years old and 13 – 14 years old

Notes to facilitators:

- *We will use an activity-based approach to the research with the Soul Buddyz Club members. This approach is based on research in childhood studies (Boyden & Ennew, 1997; O'Kane, 2010) which suggests that an approach suited to the age and stage of development of children is more likely to gather authentic data from children and young people. This approach allows young people to express their ideas in the context of their daily lives through drawings and other visual methods that allow for non-verbal responses. Note that this activity-based approach is especially useful in the context of ethics (Clacherty & Donald, 2007). Drawing allows for emotional distance - a child can draw an issue that worries them and then talk about the drawing rather than a personal experience. We also make use of hypothetical figures in this discussion as it allows for the description of an event or an opinion without the child claiming it personally.*
- *The group discussions will be facilitated by one evaluator; and one fieldworker conversant with the local language*
- *The discussion will be captured through electronic audio-recording and notes*
- *Make sure you read out information sheet to all participants and that consent to conduct and record interviews is sought from both parents/caregivers and children before running the groups. Information sheet and Consent forms are provided*
- *A group should constitute of about 8 children – no more than 10 children*
- *Estimated total time = 1 hour*

**Activity**	**Rationale**	**Questions / Probes**
Distribute food and drinks; start group activities only when the children have eaten	To make sure children have energy for the group activities and discussions	
Warm up game	To create a participatory environment and relax the children	
Facilitator: *I know you are grown up and may think drawing is for little children but It helps us to think when we draw something so I am going to ask you to do a BIG drawing together*<br><br>**Activity 1: '*What I do at SBC'***	To understand what happens at SBC – activities, lessons, challenges, successes, unintended outcomes; During analysis, assess whether SBCs are	*Use the drawing as a focus for a discussion:*<br><br>Tell me about what you have drawn<br><br>*Probes:*<br>*Get detailed descriptions of different activities and a sense of the process/approach of the club meetings*

**Instructions:** Draw all the things you do in your Soul Buddyz club here – you can draw the same or different things on this big piece of paper. Try and draw ALL the things you do."<br><br>**Materials:** pastel crayons and a largish (at least 1.5mx1.5m) piece of strong brown paper from a roll	implemented according to set objectives and design	*Information/knowledge activities:*<br><br>Do you learn anything? Get knowledge? About what?<br>How do you learn this knowledge?<br>Is it boring or fun? Why? What fun things do you do?<br>Tell me about things you do outside of school?<br>Has anything helped you as a person – inside you? Tell me about it and how it helped you.<br>Have you learned anything especially for you as a boy/girl? Tell me?<br>Do you do activities to help others? Tell me about these?<br>Are these type of activities boring or nice? Why?<br>When you wake up and know you have Soul Buddyz Club that day – how do you feel?<br>This girl here (draw a stick figure). She wants to join SBC. What would you say to her.<br>This boy (draw) wants to join. What would you say to him?<br>Point to a facilitator in one of the drawings. Who is this? Tell me about him/her.<br>Probes:<br>Facilitator gives control to SBC. Who runs the meetings? What do you do to manage/lead things?<br><br>I see you have a magazine here in this drawing. Tell me about the magazines?<br>Probes:<br>Relevance "Are the children in these magazines like<br>you?"<br>Can you read them easily?<br>Does anything ever go wrong at SBC? Do you face any problems there? Tell me about them.

		This is a SB Facilitator (draw stick figure) She wants to know what she can do to make her SBC club better! What should she do?
**Break (15 minutes)**		
Warm up Game/Song	To create a participatory environment and relax the children	
**Activity 2: *'All the hard things in our area'***<br><br>**Instructions:** Using large brown paper draw a small drawing of boy and girl (or just one if you have separated sexes). Around the small figure draw a set of bigger circles and write 'Home' in the one closest to the child; 'School' in another circle and 'village/community name' in another circle.<br>Make them big circles so they end up filling the whole paper.<br><br>**Materials:** markers, crayons, paper	To understand children's contextual challenges;<br>To understand their relationship with their parents and friends;<br>During analysis, to understand whether SBCs are relevant and respond to children needs	This is a girl/boy who lives in *Mfuleni* (name local area). She has lots of things in her life that make things difficult. Draw or write (older ones can write) the problems she faces in these different circles. Things that make her/him feel sad, frightened, worried – hard things<br><br>Tell me about what you have drawn/written.<br><br>*Probe details of issues without asking about details of personal events (ethical reason for this).*<br>*Try and keep the discussion in the third person*<br><br>So some children cannot talk to their parents about their problems? What happens? Why is this the case?<br><br>*They often need to be asked directly about home issues as they avoid these as they are difficult to talk about. Again be careful to ask in the third person e.g.*<br><br>I know some children have problems at home. Can you tell me what kind of things they could have to deal with?<br><br>*Probe communication at home and outside*

		Who do you talk to about these kind of problems? Do you talk to parents? Do they listen? Teachers? Older friends and siblings?
**Activity 3:** ***'Stickers'***<br><br>**Instructions:** Give the children some small stickers (dots) about 10 or so.<br><br>***Facilitator:*** *I want you to think about if SBCs has helped you with any of these problems you mentioned. Maybe they have not been able to make them go way but maybe they have helped you understand them or cope with them or even just talk about them. If you think SBC has helped stick a sticker next to a problem they have helped you with – even if it is in a little way. If they have not helped with anything then don't stick a sticker – no problem. Stick the dot close to the drawing so I can see where SBC has helped.*<br><br>**Materials:** Stickers; output of activity 2	To find out how relevant SBC is to the issues that children experience	*Talk about where they put the stickers.*<br><br>How has SBC helped you here?<br><br>*You can use the pattern of stickers to identify gaps as well as areas of intervention. Make sure they stick the dots close to an issue so you can see a pattern. You would use the dots as data here – though they are really just an indicative pattern.*
**Break (15 minutes)**		
Warm up game	To create a participatory environment and relax the children	
**Activity 4:** ***'SBC Materials'***<br><br>**Instructions:** Show the children the SBC materials one by one and ask the questions in third column.<br><br>**Materials:** SBC materials (Unit guides; Zones; posters…etc)	To assess children familiarity with the SBC materials;<br>To understand how the materials are used;<br>To get suggestions to improve the programme	Have you seen these?<br>Have you used them?<br>Who gave them to you?<br>How? When? How often?<br>Probe: if facilitator guided them – how?<br>What did you learn from them?<br>What do you think of them?<br>Do you have a favourite one?<br>Have you used any other materials?<br>If you were to tell Soul City Institute how to make them better what would you say?

Wrap up song<br><br>Thank participants	To wrap up	
