# Supplementary material for: The role and effectiveness of School-based Extra-Curricular Interventions on children’s health and HIV related behaviour: the case study of Soul Buddyz Clubs Programme in South Africa
Source: BMC Public Health. 2021 Dec 11;21:2259. doi: 10.1186/s12889-021-12281-8 (PMC8666065; doi:10.1186/s12889-021-12281-8)
Supplement: Supplementary file 2 — ESM 2. [file 12889_2021_12281_MOESM2_ESM.pdf]

SBC facilitator / Teacher Interview Guide

Note to facilitator:

- *Make sure the interviewee gives informed consent for interviews and for audio recording. Information sheet and consent forms are provided*
- *Interview to be captured through electronic audio recording and notes*

1. Tell me your experience facilitating Soul Buddyz Club?
    a. When did you start this work?
    b. What do you like about facilitating Soul Buddyz Club? Why?
    c. What do you dislike about facilitating Soul Buddyz Club?
    d. What are the objectives of SBC?
2. How is the attendance at the Soul Buddyz Club?
    a. What makes children to attend SBC?
    b. What discourages children from attending SBC?
3. How do you recruit children to the club?
    a. What works well?
    b. What are the challenges?
    c. Are there ways that you actively recruit vulnerable children? If so how?
4. How do you retain children in this club?
    a. What is the dropout rate?
    b. What are the reasons for dropping out?
5. How do you facilitate the Soul Buddyz Clubs?
    a. What approaches do you use?
    b. Do these approaches work? Why? Why not?
    c. Where would you improve them?
6. What materials do you use?
    a. Where do you get these materials?
    b. How do you use these materials?
    c. How effective are these materials if at all?
    d. How would you improve the materials?
7. Apart from the materials, what other resources do you use to run the SBC?
    a. Where do you get the resources?
    b. Who manages these resources?
    c. Are these resources enough? Explain
    d. What other resources would you need? Why?
8. Have you received training in facilitating SBC? If yes,
    a. When did you get this training?
    b. What were you trained on?
    c. How useful was the training?
    d. What areas do you think you need more training? Why?
9. What guidance or supervision do you get?
    a. From who?
    b. How does this happen?
    c. Are you satisfied with the support that you get? Why? Why not?
10. How do you keep track of what happens at Soul Buddyz Clubs?

a. Have you had training on the SBC mobile App?
b. Do you use the App? If not, why not?
c. What other tools do you use to track performance?
d. What in your view are the advantages of the mobile app
e. What are the difficulties with the mobile app?
f. Who do you report to? How often?
g. Do you receive feedback on your reports?

11. What is your current 'tier'/level as a club? (silver, bronze, diamond, etc)
    a. What is the best aspect of this points system?
    b. anything challenging about the points system - relative to your work?
12. Are you aware of Hands on Parenting programme in your area? If yes,
    a. What is this programme all about?
    b. How is the programme linked to Soul Buddyz Clubs if at all?
13. Which organisation do your report to?
    a. What support to you receive?
    b. What support would you like to receive?
    c. Does Soul City Institute play any role in the programme?
14. To what extent is there joint planning/collaboration between SBC facilitators and SAWs delivering HOP?
15. Have you participated in a congress? If not, why not? If yes,
    a. What is the value if any?
    b. What incentives do you receive?
16. What would you say are the successes of SBC?
17. What do you think are the challenges faced by SBC?
18. Do you see the SBC continuing after March 2019? What are the prospects?
19. What would you suggest to improve Soul Buddyz Clubs?
