# Supplementary material for: The role and effectiveness of School-based Extra-Curricular Interventions on children’s health and HIV related behaviour: the case study of Soul Buddyz Clubs Programme in South Africa
Source: BMC Public Health. 2021 Dec 11;21:2259. doi: 10.1186/s12889-021-12281-8 (PMC8666065; doi:10.1186/s12889-021-12281-8)
Supplement: Supplementary file 3 — ESM 3. [file 12889_2021_12281_MOESM3_ESM.docx]

Editor Comments:

1.      Overlap 
Thank you for revising your manuscript according to our previous requests. However it is still too high for us to proceed with.

The overlap lies between the following sections of your submission and the following publications:

•       Marinda E, Simbayi L, Zuma K, Zungu N, Moyo S, Kondlo L, Jooste S, Nadol P, Igumbor E, Dietrich C, Briggs-Hagen M. Towards achieving the 90–90–90 HIV targets: results from the south African 2017 national HIV survey. BMC Public Health. 2020 Dec;20(1):1-2.
•       <http://www.hsrc.ac.za/uploads/pageContent/10779/SABSSM%20V.pdf> 

Please note that we are unable to accept large chunks of overlap, particularly in the sections mentioned above.

Please focus on re-phrasing these sections to minimise overlap, and reference these publications appropriately.

Response: The entire quantitative research design and sample section has been rephrased to address the overlap as follows on page 6. We have also referenced the above mentioned article (Marinda et al..) in this manuscript.

Quantitative research design and sample

The quantitative data source was the fifth national survey - the South African National HIV Prevalence, Incidence, Behaviour and Communication Survey (SABSSM V, 2017), on which secondary analysis was performed to measure behavioural outcomes of the SBC programme over time. This survey was a nationally representative, cross-sectional survey of all ages living in South Africa conducted between 2017 and 2018 by the Human Science Research Council (HSRC). One thousand small area layers (SALs), as defined by Statistics South Africa, were randomly selected from a total of 84 907 SALs. (STATS-SA, 2017). Furthermore, to enrol individuals into the survey, 15 households from each SAL were selected using systematic sampling. A total of 11,776 households were ultimately included in the sample of which 82.2% of household heads consented to participate in the survey. All household members were eligible for participation in the survey. Dried Blood Spot (DBS) specimens were collected from all consenting survey respondents and samples were tested for HIV antibodies using an algorithm. ^28, 29^ For this paper, the authors used data for the 10- to 14-year-old age group to perform analyses.

The survey questionnaire, information sheets and informed consent forms were translated from English into the 10 other South African official languages: Afrikaans, isiZulu, isiXhosa, Sesotho, Setswana, Sepedi, SiSwati, Xitsonga, Tshivenda, and IsiNdebele) and piloted prior to the main fieldwork taking place. ^28^
